# An unusual S-adenosylmethionine synthetase gene from dinoflagellate is methylated

**DOI:** 10.1186/1471-2199-8-87

**Published:** 2007-10-04

**Authors:** Percy Ho, KF Kong, YH Chan, Jimmy SH Tsang, Joseph TY Wong

**Affiliations:** 1 Department of Biology, Hong Kong University of Science and Technology, Kowloon, Hong Kong SAR, China; 2 Department of Botany, University of Hong Kong, Pokfulam Road, Hong Kong SAR, China

## Abstract

**Background:**

S-Adenosylmethionine synthetase (AdoMetS) catalyzes the formation of S-Adenosylmethionine (AdoMet), the major methyl group donor in cells. AdoMet-mediated methylation of DNA is known to have regulatory effects on DNA transcription and chromosome structure. Transcription of environmental-responsive genes was demonstrated to be mediated via DNA methylation in dinoflagellates.

**Results:**

A full-length cDNA encoding AdoMetS was cloned from the dinoflagellate *Crypthecodinium cohnii*. Phylogenetic analysis suggests that the CcAdoMetS gene, is associated with the clade of higher plant orthrologues, and not to the clade of the animal orthrologues. Surprisingly, three extra stretches of residues (8 to 19 amino acids) were found on CcAdoMetS, when compared to other members of this usually conserved protein family. Modeled on the bacterial AdeMetS, two of the extra loops are located close to the methionine binding site. Despite this, the CcAdoMetS was able to rescue the corresponding mutant of budding yeast. Southern analysis, coupled with methylation-sensitive and insensitive enzyme digestion of *C. cohnii *genomic DNA, demonstrated that the AdoMetS gene is itself methylated. The increase in digestibility of methylation-sensitive enzymes on AdoMet synthetase gene observed following the addition of DNA methylation inhibitors L-ethionine and 5-azacytidine suggests the presence of cytosine methylation sites within CcAdoMetS gene. During the cell cycle, both the transcript and protein levels of CcAdoMetS peaked at the G1 phase. L-ethionine was able to delay the cell cycle at the entry of S phase. A cell cycle delay at the exit of G2/M phase was induced by 5-azacytidine.

**Conclusion:**

The present study demonstrates a major role of AdoMet-mediated DNA methylation in the regulation of cell proliferation and that the CcAdoMetS gene is itself methylated.

## Background

S-adenosylmethionine synthetase (AdoMetS) catalyzes the formation of S-adenosylmethionine (AdoMet) from methionine and ATP [1]. AdoMet participates in the regulation of a variety of cellular functions. It is a main methyl group donor and plays a central role in transmethylation reactions and the transsulphuration pathway [2]. DNA methylation is known to have regulatory effects on DNA transcription and chromosome structure. AdoMet is also involved in the biosynthetic pathway of many secondary metabolites [3,4]. It can undergo decarboxylation to generate a propylamine donor, used in the biosynthesis of polyamines [5]. Polyamines are required for cellular proliferation and may play a role in the rapid growth of bloom-forming dinoflagellates [6]. In plants, it is a precursor in the biosynthesis of ethylene [7] and serves as a methyl group donor in transmethylation of alkaloids [8]. Cell and life cycle variation in AdoMet synthetase expression has been observed in yeast and apicomplexa [9,10]. In mammals the MAT2A gene (an allele of AdoMet synthetase) is influenced by the cell cycle and is induced during liver regeneration, malignant liver transformation and T-lymphocyte activation [11]. In plants differential expression patterns for AdoMet synthetase are found in different tissues [12,13]. It is believed that expression of AdoMet synthetase can facilitate the methylation reaction and polyamine synthesis which are presumably essential during growth and morphogenesis periods.

The structures of *E. coli *and rat AdoMetS were resolved by X-ray crystallography [14,15]. Both results demonstrated an overall fold of the enzyme monomer consisting of three domains related by pseudo 3-fold symmetry: the N-terminal domain (aa 1–12 and 129–233; *E. coli *AdoMetS numbering, same below unless specified), the central domain (aa 13–101 and 234–268) and the C-terminal domain (aa 108–128 and 269–383). Two substrate binding sites are also found. A site for ATP binding between the central and C-terminal domains [16], and a methionine binding site between the central and N-terminal domain [15]. Both models posses a mobile non-visible loop (aa 103–107) linking the central domain to the C-terminal domain in close proximity to the ATP binding site. The loop is proposed to act as a gate to the site [15,17]. Analysis of rat AdoMetS has also revealed a small flexible loop (aa 251–260) near the opening of the methionine binding site. This small loop is well conserved and is directly involved in proper positioning of the methionine substrate upon binding [15].

Dinoflagellates are a distinct group with a large genome size and permanently condensed chromosomes, but interestingly lack histones and nucleosomes [18-20] Many studies have focused on the mechanism of genes transcription and DNA organization within such a huge genome in the dinoflagellate nucleus [20-24]. DNA methylation has been shown to have a role in the regulation of gene expression and chromosome structure [25,26]. Restriction endonuclease digestion analysis on ribosomal DNA of dinoflagellates shows that the genome is extensively methylated [27]. It is possible that DNA methylation may be involved in regulation of gene transcription and chromosome structure. However no full sequence of AdoMet synthetase has been reported in dinoflagellates. In this report, we have identified and characterized an AdoMet synthetase gene of the dinoflagellate *Crypthecodinium cohnii *(CcAdoMetS). Cell cycle variations of CcAdoMetS gene and protein expression are described. The state of DNA methylation status on CcAdoMetS gene and its possible feedback regulation of AdoMet synthetase expression were also investigated.

## Results

### Sequence analysis and amino acid sequence alignment

**Figure 1:**
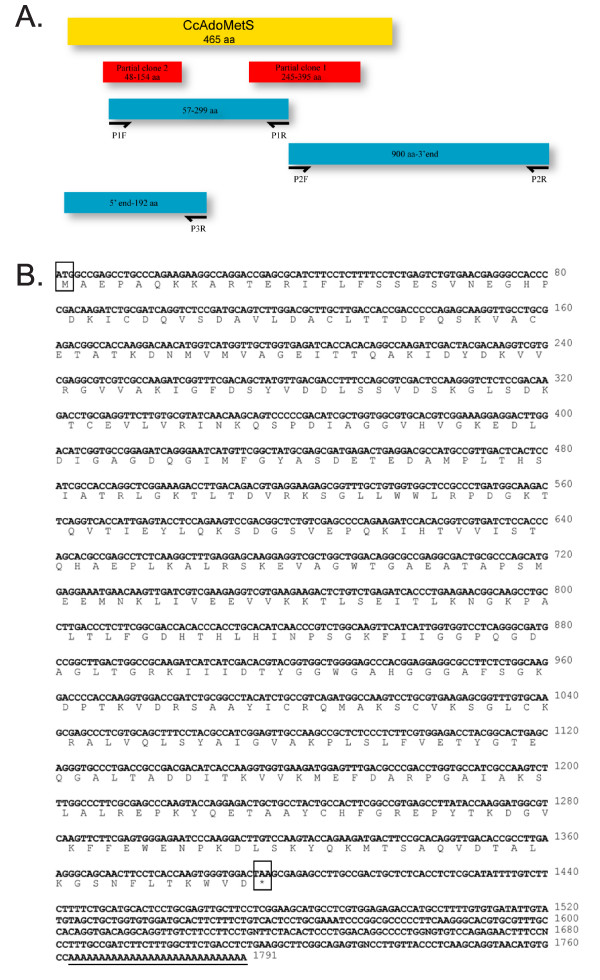
Full length of *Crypthecodinium cohnii *AdoMet synthease. (A) The sequencing strategy for AdoMet synthetase in *Crypthecodinium cohnii*. Based on two partial sequences (red rectangles) obtained from the *C. cohnii *cDNA library, the full length AdoMetS was isolated from *C. cohnii DNA *by three rounds of PCR. The center region between two *C. cohnii *clones mentioned above was isolated by PCR using P1F (5'-ATACAGCTGATGGTCATGGTA-3') and P1R (5'-CTTGCGGCCAGT CAA-3'). The 3' region of CcAdoMetS was isolated by PCR using P2F (5'-ATCATCATCGACACGTACGGTG-3') and P2R (5'-GAGAAAGGCGGACA GGTATCC-3') with C. cohnii cDNA library constructed form C. cohnii total RNA and cloned into the pDNR-LIB vector (Clontech). The 5' region was isolated by using 5'-rapid amplification of cDNA ends (BD SMARTTM RACE cDNA Amplification Kit, ClonTech) by P3R 5'-CGGCACCGATGTCCAAGTCCTCCTTT-3'. (B) Nucleotide and deduced amino acid sequences of the *Crypthecodinium cohnii *AdoMet synthetase gene. Putative start codon ATG and putative stop codon TAA are shown in the block. Poly(A) tail is underlined.

**Figure 2:**
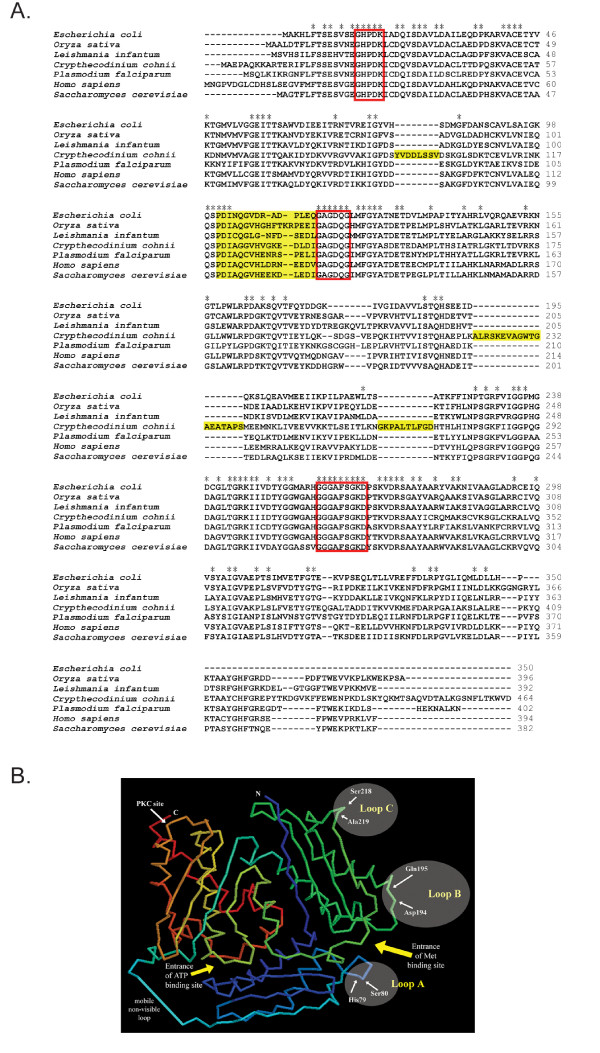
(A) Multiple alignments of AdoMet synthetase proteins. The deduced amino acid sequences of AdoMet synthetase proteins were aligned with *Plasmodium falciparum *(AAG13449), *Leishmania infantum *(AAB88448), *Saccharomyces cerevisiae *(P10659), *Oryza sativa *(CAA81481) and *Homo sapiens *(NP_000420). The identical amino acids were marked with Asterisk (*); Three conversed motifs are bracketed; The unique sequences and the flexible loops in CcAdoMet synthetase protein of *C. cohnii *are shaded. (B) Diagram showing the hypothetical positions of the three extra loops of CcAdoMetS on the basis of *E. coli *AdoMetS 3D model. The 3D model of the *E. coli *enzyme was generated form PDB file of the enzyme (Protein Data Bank ID: 1MXA) using RasMol program. The amino and carboxylic termini are labeled N and C, respectively. The three loops (Loops A, B & C) are marked with shaded eclipses on the right. The marked amino acid residues show the insertion points of the loops and are in *E. coli *enzyme numbering. The PKC site marked the equivalent position in rat enzyme for phosphorylation regulation. The mobile invisible loop is arbitrarily drawn as a blue line at the lower-left corner. Entrances of substrate binding sites are marked with yellow arrows.

Two partial clones showing high homology to AdoMetS were isolated from a *Crypthecodinium cohnii *cDNA library during random screening. Based on these fragments, a full CcAdoMetS cDNA has been obtained (Fig. 1A). Sequence analysis reveals that CcAdoMetS cDNA consists of 1395 base pairs with a putative open reading frame coding for a protein of 465 amino acids with 50.5 kDa molecular weight and 6.25 theoretical pI. The putative 3' UTR of CcAdoMetS contains 367 base pairs and a poly (A) tail (Fig. 1B). Sequence alignment of the CcAdoMetS derived from cDNA and genomic DNA has revealed that there are no introns present within the gene. The sequence of AdoMet synthetase is highly conserved among all the species tested. CcAdoMetS is approximately 50% identical to other AdoMet synthetases at the amino acid level (Fig. 2A).

CcAdoMetS shares three conserved amino acid sequences with other species. These include the conserved methionine binding motif GHPDK, the active signature hexapeptide GAGDQG, for ATP binding sites, and the conserved nonapeptide GGGAFSGKD which forms a P-loop for the phosphate-binding region (Fig. 2A). CcAdoMetS also contains the residues ^120^PDIAGGVHVGKEDLDI^135 ^(Fig. 2A) suggested to be a flexible loop which moves to gate access to the active site controlling the catalytic efficiency of AdoMetS [28]. Although AdoMetS are highly conserved among other species, the CcAdoMetS also possess three extra stretches of residues (8, 19 and 10 amino acids) at the N-terminal and middle portion of the protein (Fig. 2).

### Phylogenetic analysis

**Figure 3:**
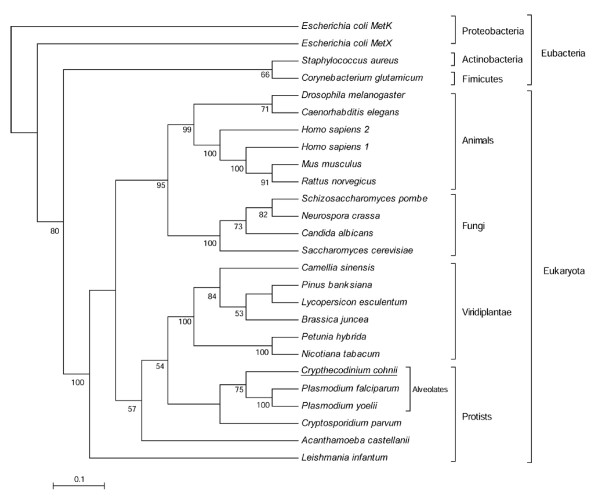
Phylogenetic tree of AdoMet synthetases. The consensus phylogenetic tree was constructed by neighbor-joining method. Numbers at each node are the percentage bootstrap value of 1000 replicates. Bootstrap values are indicated only when greater than 50%.

Phylogenetic analysis of AdoMetS shows two main distinct groupings, Eubacteria and Eukaryota (Fig. 3). Surprisingly, among the group of Eukaryota, protists and plants form a clade that is distinct from the clade of animal and fungi. CcAdoMetS is found to be immediately related to apicomplexa, a group of the subkingdom Alveolata. Further phylogenetic analyses would be required to address this plant-affiliation of both the dinoflagellate and apicomplexa AdoMetS (Fig. 3). It should be noted that both the dinoflagellate and apicomplexa contain many examples of lateral gene transfer [29]. A recent study reported on the presence of the Rubisco gene, encoding an enzyme normally involved in photosynthesis, in *C. cohnii*. Associated phylogenetic analysis in the same study supports the notion that this heterotrophic dinoflagellate was derived from a plastid bearing photosynthetic dinoflagellates, and not from early branches of heterotrophic dinoflagellates. This ancestral photosynthetic dinoflagellate was also hypothesized to be at the root of the present day photosynthetic dinoflagellates. The presence of extra sequence in the CcAdoMetS gene may thus represent an ancestral condition before the branching of the photosynthetic species [30].

### Northern blot and Western blot analysis

**Figure 4:**
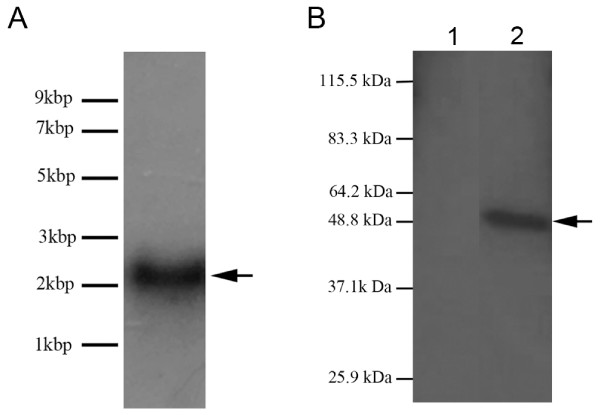
Northern and Western analysis of AdoMet synthetase. (A) AdoMet synthetase probe binds to single mRNA band of approximately 2000 bp. (B) Cell lysate of non-induced cells transformed with pQE30-AdoMet synthetase. Lane 1, non-induced pQE30-AdoMet synthetase transformed *E. coli*. Lane 2, *C. cohnii *lysate.

Northern analysis using a probe to CcAdoMetS identified to a single band with size an approximate size of 2000 bp (Fig. 4). The size of transcript was slightly larger than the cDNA however the increase in size may be explained if the 5' and 3' UTR are considered. Western analysis using a polyclonal antibody raised against CcAdoMetS protein recognized a single band with expected size of about 50 kDa (Fig. 4). These results are indicative that the obtained CcAdoMetS is a full-length clone.

### Functional complementation in yeast

**Figure 5:**
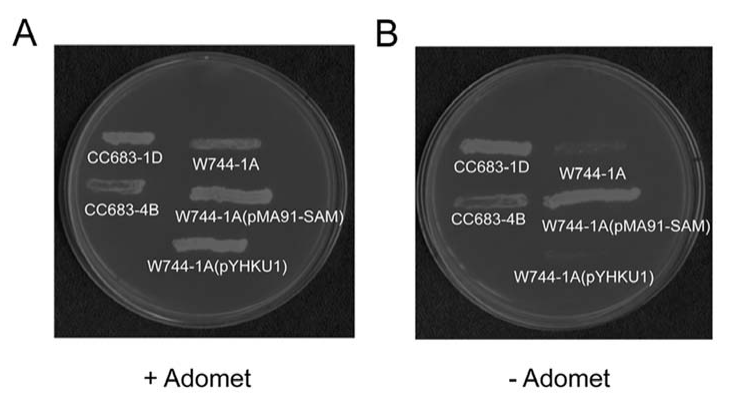
CcAdoMetS complemented AdoMetS knock-out yeast strain W744-1A. Yeast strains CC683-1D, CC683-4B, W744-1A, W744-1A(pMA91-SAM) and W744-1A(pYHKU1) were tested on medium plates supplemented with (A) 0.1 M AdoMet or (B) without AdoMet. (+) means plate supplemented with AdoMet (-) means plate without AdoMet.

Yeast strains CC683-1D, CC683-4B, W744-1A, W744-1A(pMA91-SAM) and W744-1A(pYHKU1) were tested on minimal medium plates supplemented with or without 0.1 M AdoMet. The results showed that the strains CC683-1D, CC683-4B and W744-1A(pMA91-SAM) could grow on plates while the strains W744-1A and W744-1A(pYHKU1) could only grow on the plate supplemented with 0.1 M AdoMet (Fig. 5). These demonstrated that the dinoflagellate AdoMet synthetase gene can complement the growth of the SAM defective yeast mutant W744-1A.

### CcAdoMetS transcript and protein levels during cell cycle

**Figure 6:**
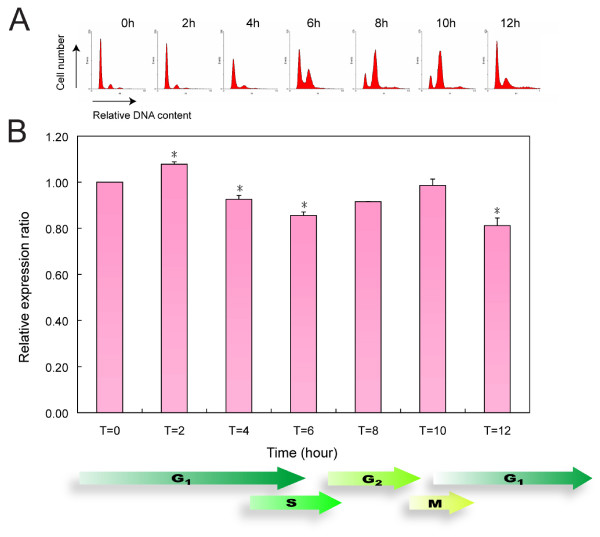
Real-Time PCR analysis of CcAdoMetS gene expression during cell cycle of *C. cohnii*. (A) Flow cytograms of synchronized *C. cohnii *culture. (B) A graph indicating AdoMet synthetase mRNA expression level of *C. cohnii *at different time points after synchronization. The data are the means ± standard deviations (n = 2). Asterisk (*) indicates significant difference between the control (T = 0) and sample values with *P *< 0.05 by Unpaired *t *test.

**Figure 7:**
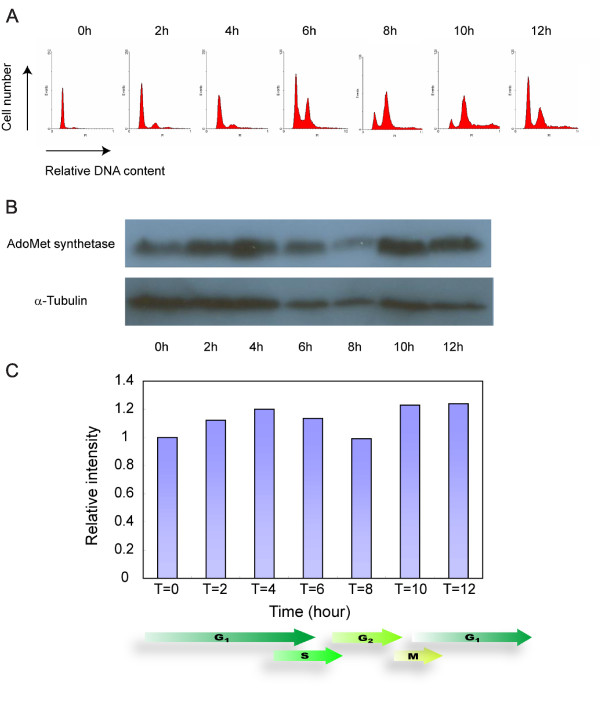
Western blot analysis of CcAdoMetS protein expression during cell cycle of *C. cohnii*. (A) Flow cytogram of synchronized *C. cohnii *culture. (B) Western blotting showing protein expression level of *C. cohnii *at different time points after synchronization. The top panel indicated AdoMet synthetase protein expression level and the lower panel indicated α-tubulin protein expression level which was used as a loading control. (C) A graph indicating the relative expression level of the AdoMet synthetase protein in *C. cohnii*. Intensities of bands were estimated by using the program "ImageJ". Values were normalized to the α-tubulin internal control and expressed relative to the T = 0 value.

The expression pattern of the CcAdoMetS mRNA transcript was analyzed by Real-Time PCR during cell cycling in synchronized cells. AdoMet synthetase mRNA transcript was found to be increased in early G1, while a decrease in transcript was observed in S phase (Fig. 6, Table 1). Western analysis indicated that the AdoMet synthetase protein also increases in late G1 phase and decreases in S phase, and again increases in the next G1 phase (Fig. 7). Both CcAdoMetS mRNA transcript and protein are expressed higher in the G1 phase. In the cell cycle of *C. cohnii*, daughter cells (after cytokinesis) spend a considerable length of time inside their mother cells [31,32]. Samples taken at T= 8 and T = 10 would have a significant portion of early G1 cells. This would account for the apparently higher level of transcripts and protein of CcAdoMet in these samples.

**Table 1:** Relative expression level of AdoMet synthetase at different time points in cell cycle

**Time (hour)**	**Relative expression ratio**
0	1.00
2	1.08 ± 0.01
4	0.93 ± 0.02
6	0.86 ± 0.02
8	0.92 ± 0.00
10	0.99 ± 0.03
12	0.81 ± 0.03

Efficiency of AdoMet synthetase gene: 1.86

Efficiency of Actin gene: 1.91

The expression level of AdoMet synthetase at all time points were compared to the expression level of AdoMet synthetase at T = 0 and normalized with actin. Each relative expression level ratio is the mean ± standard deviations (n = 2).

### DNA methylation of CcAdoMetS

**Figure 8:**
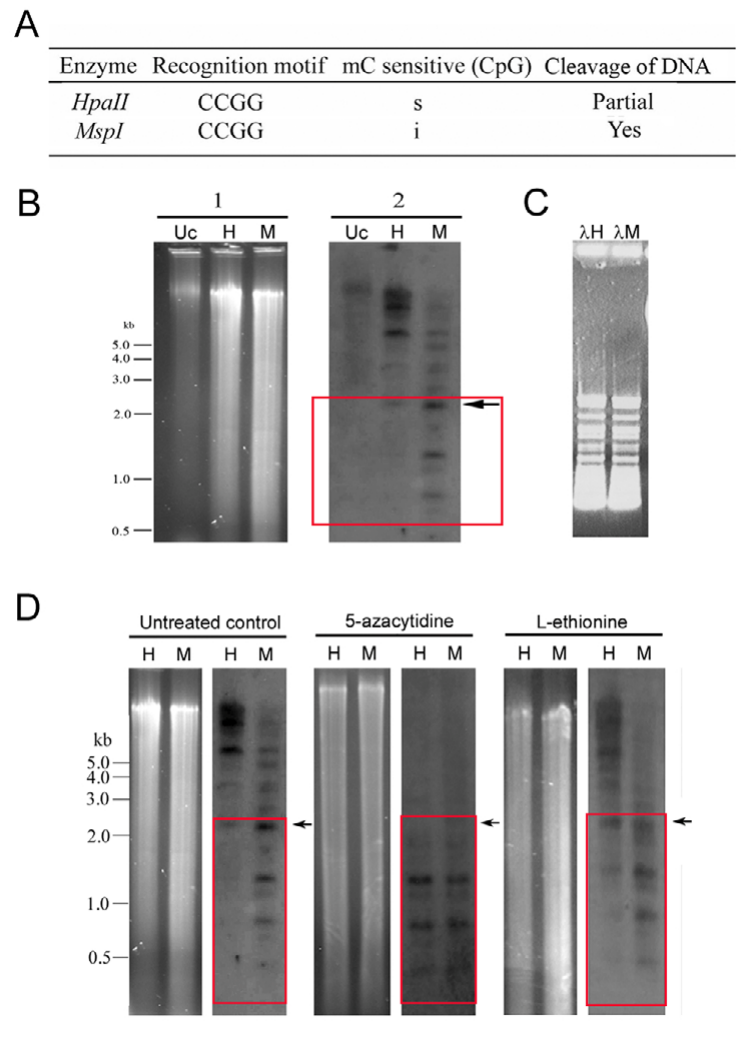
DNA methylation on CcAdoMetS gene. (A) Restriction enzyme cleavage of *C. cohnii *DNA. (B) Panel 1, Ethidium bromide stained agarose gels and panel 2, southern blotting showing restriction patterns of *C. cohnii *genomic DNA digested with isoshizomeric enzymes *HpaII *and *MspI *recognizing CCGG sequences. Lane Uc was uncut DNA. Lane H and M showed the pattern of restriction after DNA was treated with *HpaII *and *MspI *respectively. Absence of fragments smaller than around 2 kbp was observed in Lane H (Arrow). (C) The presence of interference within digestion was tested by treating with bacteriophage λDNA restriction enzyme with *HpaII and MspI *restriction enzymes. Lane λH and λM showed the restriction pattern of *HpaII and MspI *respectively. Lane λUc was uncut control λDNA. (D) Southern blot analysis of *C. cohnii *DNA after treated with DNA methylation inhibitors 5-azacytidine and L-ethionine. Ethidium bromide stained agarose gels and southern blotting showing the restriction pattern of DNA by *HpaII *(H) and *MspI *(M) without any treatment; after treating with 200 μM 5Aza and after treating with 1.2 mM L-Eth. Presence of fragments smaller than around 2 kbp was observed in *HpaII *digested DNA after treated with 5Aza and L-Eth (Arrow).

Methylcytosine has been reported within the *C. cohnii *genome, mainly at CpG residues [27]. To investigate whether the AdoMet synthetase gene is methylated genomic DNA digested with the restriction enzymes *HpaII *or *MspI *was hybridized to a labeled AdoMetS probe. *HpaII *recognizes and cleaves CCGG sequences but digestion is prevented when the internal cytosine residues within the CCGG sequences is methylated. However *MspI*, the isoschizomer of *Hpa II*, cleaves the same DNA sequence whether the sequence is methylated or not (Fig. 8A). Inhibition of digestion of the gDNA by the methylation sensitive enzyme *Hpa11 *was observed. Only large DNA fragments were observed after DNA treating with *HpaII*. In contrast, small DNA fragments smaller than 2 kb were produced after digesting with *MspI *indicated by arrow on Fig. 8B. This is indicative of DNA methylation sites on CCGG residues within the CcAdoMetS gene. To eliminate the possibility that inhibition of digestion was due to the presence of interference during digestion, bacteriophage λ DNA was treated with *HpaII *and *MspI *restriction enzymes. All of the enzymes could cleave λ DNA to completion. (Fig. 8C).

Two DNA methylation inhibitors, 5-azacytidine (5Aza) and L-ethionine (L-Eth) were used to confirm that the inhibition of digestion by methylation sensitive enzyme was caused by the presence of cytosine methylation on AdoMetS gene. 5Aza is a base analogue which can be incorporated into DNA during replication. It inhibits DNA methyltransferase and prevents the transfer of a methyl group onto the newly synthesized DNA strand [33]. Southern analysis indicated that the genomic DNA of 5Aza-treated (200 uM) cells was more susceptible to DNA methylation- sensitive enzymes. Fragments smaller than 2 kbp were observed in both *HpaII *and *MspI *digested DNA after treating the cells with 200 μM 5Aza when compared with the control (Fig. 8D). L-Eth is an analog of methionine and produces S-adenosylethionine which competes with AdoMet in transmethylation reactions [34,35]. After L-Eth treatment, Southern analysis shows the digestibility of gDNA by *HpaII *increased. Hybridized fragments smaller than 2 kbp were observed in *HpaII *digested gDNA. However, the increased digestibility of AdoMetS gene when using the L-Eth methylation inhibitor was less than found for the 5Aza inhibitor. It can be assumed then that the overall reduction of methylcytosine within CCGG sequence in AdoMetS gene by L-Eth was less then 5Aza (Fig 8D). The use of the two DNA methylation inhibitors has confirmed that inhibition of *HpaII *digestion was the result of DNA methylation on CCGG residue (Table 2).

**Table 2:** Inhibition of DNA methylation of AdoMet synthetase by 5-azacytidine and L-ethionine

	**The degree of digestibility of CcAdoMets gene**		**Inhibition of DNA methylation of CcAdoMets gene**
	***HpaII***	***MspI***	
		
**Untreated**	*	****	/
**5-azacytidine treatment**	*****	*****	*****
**L-ethionine treatment**	***	****	***

* Represents the degree of digestibility and inhibition of DNA methylation

Southern blotting showing the restriction pattern of *C. cohnii *DNA by *HpaII *and *MspI *without any treatment; after treating with 200 μM 5Aza and after treating with 1.2 mM L-Eth. Higher the digestibility of CcAdoMetS gene by *HpaII *indicated higher degree of inhibition of DNA methylation.

### Effects of DNA methylation inhibitors on cell cycle progression

**Figure 9:**
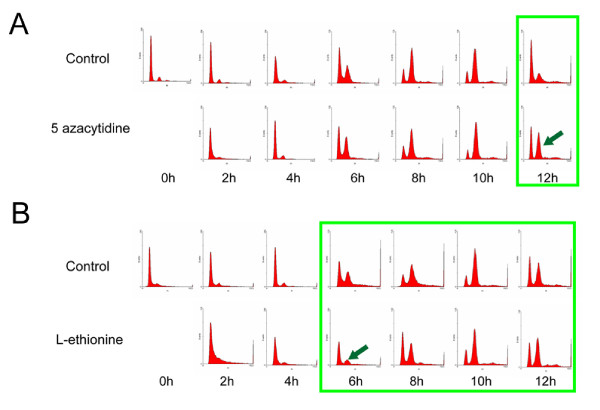
Effects of DNA methylation inhibitors on *C. cohnii *cell cycle progression. (A) Flow cytograms of synchronized *C. cohnii *culture. Upper and lower panel is the flow cytograms of control and cells treated with 200 μM 5-azacytidine respectively. Arrows indicated the delay at the exit of G2/M phase. (B) Flow cytograms of synchronized *C. cohnii *culture. Upper and lower panel is the flow cytograms of control and cells treated with 1.2 mM L-ethionine respectively. Arrows indicated the delay at the entry of S phase. Green blocks indicate delay of cell cycle.

DNA methylation sites present on CcAdoMetS, may be involved in the control of cell cycle progression. To investigate this the two methylation inhibitors, 5Aza (200 μM) and L-Eth (1.2 mM) were added to synchronized *C. cohnii *cells and cells harvested every two hours for ten hours. Flow cytometric analysis revealed a delay at the exit of G2/M phase when the cells were treated with DNA methylation inhibitor 5Aza. However, a cell cycle delay was observed at the entry of S-phase when treating the cells with L-Eth (Fig. 9).

### CcAdoMetS protein expression after treatment with DNA methylation inhibitors

**Figure 10:**
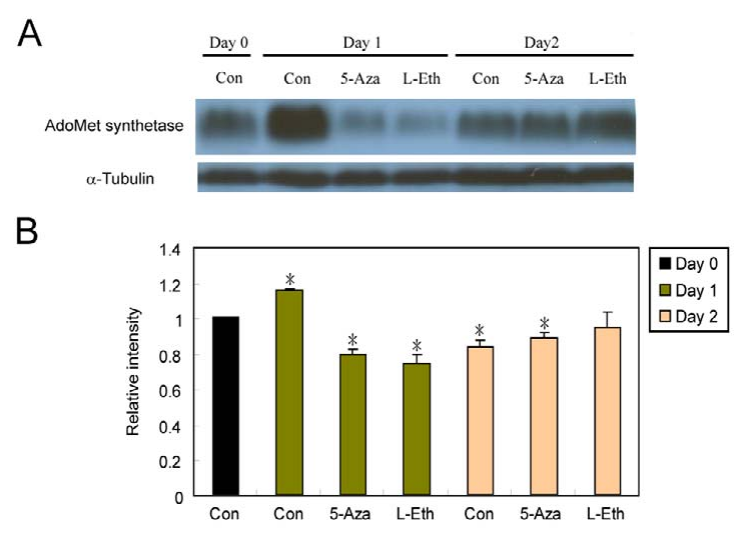
Western blot analysis of CcAdoMetS protein expression after treating with 5-azacytidine and L-ethionine. (A) Western blotting showing protein expression level of *C. cohnii *on different days without any treatment; after treating with 200 μM 5Aza and after treating with 1.2 mM L-Eth. The top panel indicated AdoMet synthetase protein expression level and the lower panel indicated α-tubulin protein expression level which was used as a loading control. (B) A graph indicating the relative expression level of the AdoMet synthetase protein in *C. cohnii*. Intensities of bands were estimated by using the program "ImageJ". Values were normalized to the α-tubulin internal control and expressed relative to the value of control on day 0. The data are the means ± standard deviations (n = 2). Asterisk (*) indicates significant difference between the control on day 0 and sample values with P < 0.05 by Unpaired t test.

The effect of 5Aza and L-Eth treatment on AdoMet synthetase protein expression was also analyzed but in asynchronized cells. Two methylation inhibitors, 5-Aza (200 μM) and L-Eth (1.2 mM) were added to asynchronized *C. cohnii *cells at day 0 independently and untreated *C. cohnii *cells were used as a control. Cells were collected at day 0, 1 and 2 and were subjected to western analysis. Interestingly, in the untreated control, higher expression of CcAdoMetS protein was observed at day 1 compared to the control at day 0. After treatment with 5Aza or L-Eth, a significant reduction of CcAdoMetS protein expressions was observed. CcAdoMetS protein level in both treated and untreated cells returned to similar level at day 2 (Fig. 10).

## Discussion

AdoMetS has a central role in metabolism of all cells. AdoMetS is highly conserved and appears to maintain the same overall structure in different species including CcAdoMetS. Sequence alignment of various AdoMetS reveals that CcAdoMetS processes three extra loops, which insert between the positions equivalent to *E. coli *AdoMetS H80-S81 (loop A; 8 aa), D195-Q196 (loop B; 19 aa) and S219-A220 (loop C; 10 aa) (Fig. 2). According to the established crystal structure of *E. coli *AdoMetS, the first two loops (A and B) would be located at the opening of methionine binding site, and the loop C would be close to the cleft between the N- and C-terminal domains (Fig. 2B). Based on their sizes and positions, we postulate that loops A and B may pose some effects on substrate binding or product release of the methionine binding site and possibly the reaction kinetics. Loop C however is not positioned close to the substrate binding sites nor regions for dimerization or tetramerization [15]. On the opposite side of the N-/C- terminal domain cleft of the rat AdoMetS, there is a protein kinase C site (T342 at the N-temrinal end of helix 10; rat AdoMetS numbering) which is responsible for regulation of enzyme activity and dimerization through PKC phosphorylation [36]. This threonine residue is conserved in human, *Plasmodium *and the dinoflagellate AdoMetSs (T378), but not in yeast and plant enzymes (Fig. 2A). It is possible that this N-/C- terminal cleft, including the additional loop C in CcAdoMetS, is a potential regulatory region subjected to post-translational regulation systems.

The present study of CcAdoMetS revealed that both the transcript and protein level were relatively higher at G1. In the dinoflagellate *Pyrocystis lunula*, microarray analysis suggested that a putative AdoMet synthetase transcript level was 2.2 times higher in the early light phase when compared to that of early dark phase [37]. Conversely, S-adenosylhomocyteine (AdoHcy) hydrolase is downregulated during G1 phase in dinoflagellate *Alexandrium fundyense *[38]. AdoHcy hydrolase is the enzyme that catalyzes the conversion of AdoHcy which is formed after AdoMet transferring methyl group to its acceptor, into methionine and re-enter the methyl-cycle. Accumulation of AdoHcy is showed to be a target for inhibition of cellular methyltransferases and can influence methylation processes [39]. It is apparent that enzymes involved in cellular methylation are regulated in a cell-cycle dependent manner. The G1 expression of AdoMetS is also consistent with late G1 synthesis of saxitoxin [3,4] in dinoflagellate *Alexandrium fundyense *[40]. For toxic bloom-forming dinoflagellates, AdoMet synthetase may take part in rapid growth and toxin formation. The level of cell-cycle variation of the CcAdoMetS transcripts, from 0.8 to 1.2 (Fig. 6, 7), is much lower than the previously reported (2.2 fold in *Pyrocystis lunula*). Since *C. cohnii *is a heterotrophic species, this may reflect that the previous reported variation in photosynthetic species could be contributed by light-dark cycle. Plastid-encoded psbA proteins were reported to be light-regulated in dinoflagellates[41].

Upon inhibition of DNA methylation, an increase in digestibility of *C. cohnii *genomic DNA by methylation sensitive enzyme was observed. These suggest that methylcytosine was present on CCGG residues of CcAdoMetS gene. In the present study, treating synchronized *C. cohnii *cells with 5Aza and L-Eth, delayed cell cycling and changed AdoMetS protein expression. 5Aza treatment caused a delay at the exit of G2/M in *C. cohnii *cell cycle. One of the possible reasons is that 5Aza induced DNA hypomethylation which may prevent chromosome compaction. Moreover demethylated DNA may affect DNA-protein interactions, e.g. alternation of DNA interaction of structural maintenance of chromosomes (SMC) family proteins and thereby alters chromosome compaction [42]. All these may influence the mitosis process and delay entry into G2/M. 5Aza can also be an inducer of chromosome damage [43], which may activate cell cycle checkpoint and delay the progress of cell cycle [44].

Another DNA methylation inhibitor L-Eth caused a delay at the G1/S entry. Such a delay was also observed in human lymphocytes cell when treated with L-Eth [45]. It is believed that DNA methylation is critical to the initiation of DNA synthesis. Some studies reported that the origin of the replication site of *E. coli *DNA is heavily methylated [46] and DNA methylation could affect the initiation of DNA replication at some origins in mammalian cells [47]. Dinoflagellates genomes are well known to have both prokaryotic and eukaryotic properties [48,49], it is possible that DNA methylation may have a role in the regulation of DNA replication.

For the fast growing log phase *C. cohnii *cells, higher CcAdoMetS protein expression was observed at day 1. DNA methylation inhibitors 5Aza and L-Eth can cause DNA hypomethylation [33-35] which is normally associated to the activation of gene expression [25,26], and thus higher expression of CcAdoMetS protein may be expected after treatments with these inhibitors. However lower expression of CcAdoMetS protein was observed after treatment with either 5Aza or L-Eth. Our data implicates that methylation on CcAdoMetS gene may not be the only way to control its expression (Fig. 10). Further work is required to delineate the exact mechanisms of regulation. A knowledge of the regulatory mechanism of gene expression in dinoflagellates may, in turn, lead the way to dissect molecular mechanism of cell-cycle gating by the circadian rhythm[50].

## Conclusion

CcAdoMet is the first full-length AdoMet synthetase that has been reported from dinoflagellates. This dinoflagellate AdoMetS has three extra loops when compared to other members of this highly conserved gene family. Despite this, the CcAdoMetS was able to complement the corresponding mutant in budding yeast (Fig. 4). The present study demonstrates that the CcAdoMetS gene is itself methylated. Inhibitor studies and cell-cycle expression pattern suggest that CcAdoMet-mediated DNA methylation has a role in the regulation of cell proliferation.

## Methods

### Cell culture, cell cycle synchronization and flow cytometric analysis

*Crypthecodinium cohnii *strain (Biecheler) 1649 was obtained from the Culture Collection of Algae, University of Texas. Cells were cultured in MLH medium [51] in the dark at 28°C. Synchronization was carried out as described previously [52]. Synchronized cells were harvested every two hours and subjected to flow cytometric analysis. Cells were fixed in 70% ethanol overnight and resuspended in PBS, pH7.4 and 5 μg/ml RNaseA. After incubation at 37°C for 1 hour, samples were stained with 0.025 mg/ml propidium iodide (PI) and subjected to measurements using Fluorescent Activated Cell Sorter (FACS) on a Becton-Dickinson Vantage flow-cytometer. Data was analyzed using the software WinMDI (version 2.8).

### Cloning and sequencing of the S-adenosylmethionine synthetase

Random sequencing of a *C. cohnii *cDNA library identified two clones with significant sequence homology to AdoMet Synthase of different organisms. Using this sequence information and 5' RACE (BD SMART™ RACE cDNA Amplification Kit, ClonTech) the full sequence was obtained (Fig. 1). The full length AdoMetS cDNA encoding for the ORF (1–1395 bp) was amplified by PCR using the sense primer 5'-TATGGATCCATGGCCGAGCCTG-3' (including a BamHI restriction site) and antisense primer 5'- ATAAAGCTTTTAGTCCACC CACTTGGTG-3' (including a HindIII restriction site) and cloned into pGEM^®^-T Easy Vector (Promega) and sequenced (ABI Prism BigDye Terminator v3.0 Ready Reaction Cycle Sequencing Kit).

### Sequence alignment and phylogenetic analysis

CCAdoMet and an additional twenty-five AdoMet synthetase sequences of different species including bacteria, protists, fungi, plant and animal were retrieved from the NCBI sequence database and aligned using the CLUSTALW program in BIOEDIT [53]. Phylogenetic sequence analysis was carried out using PHYLIP, version 3.5 [54]. Using the PRODIST program with Jones-Taylor Thornton matrix, one thousand bootstrap replicates were generated and distance calculated by the neighbor-joining method. Phylogenetic trees were drawn using MEGA2 [55].

### Expression of AdoMetS and polyclonal antibody production

CcAdoMetS from 39 bp-1395 bp was amplified by PCR with sense primer 5'-TATGGATCCACGT TCCTGTTCTCCTC-3' (including a *BamHI *restriction site) and antisense primer 5'-ATAAAGCTTTTAGTCCACCCACTTGGTG-3' (including a *HindIII *restriction site). The amplified fragment was cloned into expression vector pQE30 (QIAexpress System, QIAGEN) and transformed into *Escherichia coli *SG10039. Cells were grown at 37°C until OD_600 _0.6. and then induced using 1 mM isopropyl into-β-galactoside (IPTG) for 4 hours. Bacteria were harvested and resuspended in denaturing buffer (100 mM NaH_2_PO_4_, 10 mM Tris Cl and 8 M Urea). The 6xHis tagged-recombinant protein was then purified by Ni^2+ ^nitrilotriacetic acid (Ni-NTA) affinity chromatography under denaturing conditions using QIAexpressionist™ protein expression and purification system (Qiagen Corporation). After dialysis, the recombinant protein with 6xHis tag was used to produce a polyclonal antibody against the recombinant CcAdoMetS, according to established protocols [56]. The antibodies were purified by affinity purification as method previously described [57,58] and cleaned antibodies were then used for western blotting.

### Functional complementation in yeast

For the yeast complementation, a full CcAdoMetS gene (1.3 kb) with BamHI attached at both ends was amplified by sense primer (5'-GTATGGATCCATGGCCGAGCCTG-3') and antisense primer (5'-GATTGGATCCTTAGTCCACCCACTTGGTG-3') from *C. cohnii *cDNA. The amplified fragments were then cloned into pMA91 vector, which contains LEU2 gene that was required for yeast growth in leucine-deficient growth media.

Since *Saccharomyces cerevisiae *AdoMetS-deficient double-mutant strain W744-1A has a LEU2 gene, for selection of clones harboring the vector only was used as a negative control for yeast complementation. pYHKU1 was constructed by replacing the LEU2 gene marker of the pMA91 vector with another marker URA3 from pYES2 yeast vector. The yeast W744-1A was separately transformed with pYKU1 and pMA91-SAM by a modified lithium acetate method [59]. Cell (W744-1A) transformed with pYHKU1 was used as a negative control. Other yeast strains used were CC683-1D (sam1, SAM2), CC683-4b (SAM1, sam2) and W744-1A (sam1, sam2). All the yeast stains were tested on minimal medium plates supplemented with or without 0.1 M AdoMet. Minimal medium plates containing 2% glucose, 0.78% yeast nitrogen base with the presence or absence of amino acids.

### Restriction endonucleases and Southern blot analysis

Mid-log phase *C. cohnii *cells were collected and genomic DNA isolated using cetyltrimethylammonium bromide (CTAB) buffer [60]. Purified DNA (15 μg) was restricted by 5U/μg restriction enzymes (*Msp*I and *Hpa*II). Digested genomic DNA was fractionated on 1.2% agarose gels and blotted onto nylon membrane (GeneScreen) Membranes were then hybridised with a 700 bp CcAdoMetS probe, corresponding to nucleotides 222 bp-922 bp, labelled using the ECL Direct Nucleic Acid Labelling System (Amersham Biosciences).

### RNA extraction and Northern blot analysis

Total RNA was isolated from *C. cohnii *cells by LiCl/Urea precipitation. For northern blot analysis, extracted total RNA was electrophoresed on a 1% formaldehyde agarose gel (1% agarose, 18% formaldehyde, 1× MOPS) and transferred to nylon membrane (GeneScreen) The RNA was hybridized with a 589 bp CcAdoMetS probe, corresponding to 900 bp-1489 bp, labelled with [α-^32^P] dCTP using the Rediprime II random primer labeling system (Amersham Biosciences).

### Real time RT-PCR

First-strand cDNA was synthesized in a 20 μl reaction volume containing 1 μg of total RNA, 200 units of SuperScriptTM II Reverse Transcriptase, 10 mM dNTPs, 40 units of RNase inhibitor and 2 pmole gene specific primer (AdoMet synthetase gene, 5'-GACACCATCGCCAGAGTCCATCA-3' or Actin gene, 5'-GACACCATCGCC AGAGTCCATCA-3'). Total RNA was denatured for 5 mins at 65°C and mRNA was reverse transcribed at 42°C for 50 mins before heat-inactivation for 15 mins at 70°C. Real time RT-PCR was performed with a LightCycler (Roche) using LightCycler FastStart DNA Master PLUS SYBR Green I (Roche). Each PCR was performed in a 20 μl reaction with 4 μl 5× master mix, 10 μM forward primer (CcAdoMetS gene, 5'-CCTCACGTCTGTCAAGGTCT-3'or reference gene, actin 5'-CTTCAACGTCCCCGCCATGTAC-3'), 10 μM reverse primer (AdoMet synthetase gene, 5'-GACACCATCGCCAGAGTCCATCA-3' or reference gene actin, 5'-GACACCATCGCCAGAGTCCATCA-3'). The thermal cycling conditions used: 95°C for 10 mins; followed by 45 cycles of denaturation at 95°C for 10s, optimal annealing temperature (CcAdoMetS gene, 57°C; Actin gene, 65°C) for 10s and extension at 72°C for 10s. Measurements were carried out in duplicates. Data was then normalized using actin as the reference gene and relative expression calculated using the method described by the PFAFFL group [61,62]. The data are the means ± standard deviations (n = 2). Asterisk (*) indicates significant difference between the control (T = 0) and sample values with *P *< 0.05 by Unpaired *t *test with GraphPad software[63].

### Protein extraction and western blotting

*C. cohnii *cells were resuspended in denaturing buffer (100 mM NaH_2_PO_4_, 10 mM Tris Cl and 8 M Urea) and boiled for 5 mins. Proteinase inhibitors (20 μg/ml leupeptin, 20 μg/ml aproptinin, 2 μg/ml pepstatin A and 1 mM PMSF) were then added and the cells sonicated. Lysate was collected and protein concentration quanitified by the BioRad DC protein assay kit (Bio-Rad). SDS-PAGE analysis was carried out in 12% polyacrylamide gels containing acrylamide and bis in a ratio of 30:0.8. Western transfer to polyvinylidene fluoride (BioTraceTM) membrane was carried out by semi dry transfer technique [55]. The transferred membrane was blocked with 3% no fat milk powder in TBS-T and was then probed by the primary polyclonal anti-CcAdoMet synthetase overnight at 4°C. The blots were developed using horse radish peroxidase conjugated secondary antibodies and a ECL western blotting detection system (Amersham). α-tubulin was used as a loading control.

### Inhibition of DNA methylation using chemical inhibitors

The DNA methylation inhibitors 5-azacytidine (5Aza) at 200 μM and L-ethionine (L-Eth) at 1.2 mM were dissolved in MLH media before addition to synchronized or asynchronised cells [33-35]. To confirm the inhibition of digestion by methylation sensitive enzyme is due to presence of methylcytosine in CCGG residues the inhibitors were added to asynchronized *C. cohnii *cells for two days before cells were harvested and gDNA digestion completed as previously. To study the effects of inhibition DNA methylation on cell cycle progression inhibitors were added to synchronized C. cohnii cells at T = 0 and cells harvested every two hours for twelve hours before analysis by FACs. To study effects of inhibition of DNA on AdoMetS protein expression the inhibitors were added to asynchronised log phase cells. Cells were harvested at day 0, 1 and 2, protein extracted and subjected to Western analysis using the polyclonal antibody to CcAdoMetS. Values were normalized to the α-tubulin internal control and expressed relative to the value of control on day 0. The data are the means ± standard deviations (n = 2). Asterisk (*) indicates significant difference between the control on day 0 and sample values with P < 0.05 by Unpaired t test with GraphPad software [63].

## Competing interests 

The author(s) declares that there are no competing interests.

## Authors' contributions

PH carried out the molecular biological studies, participated in the sequence alignment and drafted the manuscript. KFK carried out the yeast complementation. JSHJ and JTYW participated in the design of the study. JTYW conceived of the study, and participated in its design and coordination and helped to draft the manuscript. All authors read and approved the final manuscript.
